# Advance Analysis of the Obtained Recycled Materials from Used Disposable Surgical Masks

**DOI:** 10.3390/polym16070935

**Published:** 2024-03-29

**Authors:** Alen Erjavec, Julija Volmajer Valh, Silvo Hribernik, Tjaša Kraševac Glaser, Lidija Fras Zemljič, Tomaž Vuherer, Branko Neral, Mihael Brunčko

**Affiliations:** 1 Faculty of Mechanical Engineering, University of Maribor, Smetanova cesta 17, 2000 Maribor, Slovenia; julija.volmajer@um.si (J.V.V.); tjasa.glaser@um.si (T.K.G.); lidija.fras@um.si (L.F.Z.); tomaz.vuherer@um.si (T.V.); branko.neral@um.si (B.N.); mihael.bruncko@um.si (M.B.); 2 Faculty of Electrical Engineering and Computer Science, University of Maribor, Koroška cesta 46, 2000 Maribor, Slovenia; silvo.hribernik@um.si

**Keywords:** mechanical recycling, disposable surgical mask, morphology, surface properties, mechanical properties, nonwoven materials, PPE

## Abstract

The production of personal protective equipment (PPE) has increased dramatically in recent years, not only because of the pandemic, but also because of stricter legislation in the field of Employee Protection. The increasing use of PPE, including disposable surgical masks (DSMs), is putting additional pressure on waste collectors. For this reason, it is necessary to find high-quality solutions for this type of waste. Mechanical recycling is still the most common type of recycling, but the recyclates are often classified as low-grade materials. For this reason, a detailed analysis of the recyclates is necessary. These data will help us to improve the properties and find the right end application that will increase the value of the materials. This work represents an extended analysis of the recyclates obtained from DSMs, manufactured from different polymers. Using surface and morphology tests, we have gained insights into the distribution of different polymers in polymer blends and their effects on mechanical and surface properties. It was found that the addition of ear loop material to the PP melt makes the material tougher. In the polymer blends obtained, PP and PA 6 form the surface (affects surface properties), while PU and PET are distributed mainly inside the injection-molded samples.

## 1. Introduction

Personal protective equipment (PPE), such as face masks, gloves, goggles, clothing and aprons, is the most important type of protection for individuals when they are exposed to pathogens and infections in the workplace [1,2]. PPE is defined by the World Health Organization (WHO) as equipment and/or clothing used by personnel to provide protection against biological agents and to reduce individual exposure [3]. When we talk about PPE, we certainly think first of its use in healthcare, which represents approximately one-third of all PPE use. However, the remaining two-thirds are spread across many areas of use, such as manufacturing, construction, the chemical industry, pharmacy, transportation, mining, the food industry and others. With the year 2019 and the outbreak of the viral disease COVID-19 [4], the use of PPE expanded from use in the workplace (business use) to use in households (private use). This led to a huge increase in demand for PPE, and, consequently, an increase in production. In the five-year period between 2016 and 2020, the annual growth of the global PPE market was 6.5%, and the value of this market increased from USD 40 billion to USD 58 billion. During this period, the COVID period saw an increase in annual growth to 20% and had already reached USD 68.5 billion in 2022 [2,5]. According to various market forecasts, the production of PPE made from nonwovens will continue to increase despite the end of the pandemic [5,6]. The increasingly strict laws in the area of Employee Protection in various industries, in which the protection of the individual is becoming more and more important, will certainly play a major role in this. The fact that PPE made from nonwovens is a disposable product that is disposed of after a short period of use is a major cause for concern and poses a serious threat to the environment [7,8,9,10,11]. One of the most sought-after products in the PPE sector in recent years has been the disposable surgical mask (DSM). Not only were DSMs mandatory equipment in the healthcare sector, but governments around the world made the use of DSMs mandatory in public places [9]. The increase in demand led to an increase in production. The higher number of DSMs in circulation increases the pressure on waste collectors. The consequence of increased waste generation is reflected in increased waste losses that can be detected in the natural environment [7,12]. As has been pointed out in many studies, DSMs in the natural environment cause serious pollution by the leaching of chlorine, micro and nanoplastics [7,8,10,11,13,14].

The results of all the aforementioned research have led to an intensive search for solutions in the field of DSM waste treatment. Researchers around the world are looking for possible solutions in this area, from different types of disinfection with the aim of reuse to different types of recycling—mechanical recycling, thermomechanical recycling and pyrolysis—to obtain usable secondary materials [15,16,17,18,19,20,21,22,23,24].

The recycling of waste DSMs presents a real challenge, as they are made of different materials. They consist of five main components, namely ear loops, nose wire, inner and outer nonwoven layers and a middle, filtering melt-blown layer [10]. The outer, inner and middle layers can also be considered one part of the DSM, as they are made mainly of polypropylene (PP). For the ear loops, the material used in the manufacturing process varies much more. Materials such as polyamide 6 (PA 6), also known as nylon 6, polyurethane (PU), also known as elastane, and polyethylene terephthalate (PET) have been identified. Some manufacturers even use a so-called core filament, in which an elastic plane core is coated with a nonwoven PP fabric [10,16]. In investigating different types of nose wires, three different models have been detected: a nose wire with one metal wire coated by PP, a nose wire with two metal wires coated by PP, and a nose wire without metal wire—a plastic stripe made of PP [10]. The ones with metal wires cause another problem in terms of recycling and should be separated from other parts of DSMs before recycling.

In the field of recycling, primary recycling is the most desirable as it follows the principle of closing the loop and the recovered materials do not lose their primary value, as is the case with secondary recycling (downcycling), or tertiary (chemical depolymerization) and quaternary (energy recovery) recycling [25,26]. Mechanical recycling can be classified as both primary and secondary recycling. It involves the processing of plastic waste by shredding it into small granules, which can then be melted down and processed into secondary material suitable for new products [27,28].

In this study, the mechanical recycling approach was identified as the most suitable recycling option for waste DSMs. Compared to previously published articles, this study used already used DSMs that came from public use, so the sample was much more heterogeneous and mimicked real waste samples. The samples were pretreated and then extruded. Test specimens were produced from the resulting granulate by injection molding. The focus of the properties investigated was not only on the mechanical and thermal properties of the obtained material, but also on the surface properties and morphology of the test specimens produced. These properties, in addition to the mechanical properties, are of serious importance in order to find the right end application of the obtained material and to prevent it from losing its original value.

The objective of the research work is to show that used DSMs can be a secondary raw material from which a new material with almost identical mechanical, thermal and chemical properties can be obtained without using petroleum derivatives and without having to accept the negative effects of improperly disposed of DSMs on the environment. This is a step toward the concept of a circular economy.

## 2. Materials and Methods

### 2.1. DSMs’ Recycling Process and Test Samples’ Production

The samples of used DSMs were collected from public use for 6 months during the pandemic. The samples consisted of disposable surgical masks in different colors, some with printed patterns, and in different sizes—from child to adult sizes. The samples were isolated for 3 weeks, and then a manual separation was performed to remove other types of face masks that were present in the collected sample (types FFP2, FFP3, etc.). The rough estimate of the total DSMs collected and sorted was between 7000 and 8000 pieces, which were disassembled manually to remove the iron nose wires and to separate the ear loops from the three-layered part.

The ear loops and the three-layer part were cut by hand, and then ground separately using the Pulverisette 25 Power Cutting Mill with cyclone sample extraction (Fritch GmbH, Laterns, Austria). A 0.5 mm trapezoidal perforation sieve was used during the cutting process.

Two different milled samples were collected, namely the three-layered part (r3L) and the ear loops (rEL). The third sample was prepared by mixing both in the weight ratio as they are represented in the DSM—14% milled ear loops and 86% milled three-layered part (rDSM).

All 3 samples were extruded using a twin screw extruder, ThermoFisher Scientific process 11 (ThermoFisher Scientific Inc., Waltham, MA, USA) under the conditions given in Table 1. The extrusion screw profile consists of eight zones: a feeding/conveying zone; a mixing/conveying zone; a conveying zone; a mixing/conveying zone; a conveying zone; a mixing/conveying zone; a conveying/venting zone; and a discharging zone.

The extruded filaments of all 3 samples were pelletized into pallets that were used for injection molding of the test samples with the HAAKE™ MiniJet Pro Piston Injection Molding System (ThermoFisher Scientific Inc., Waltham, MA, USA). The mold used for the production of the test samples to perform the majority of tests was 80 mm × 10 mm × 4 mm. Two other molds were used to produce samples for the tensile and bending tests. The mold used to produce the tensile test specimens was according to the ASTM D638 IV Standard [29], and the specimens’ dimensions were 115 mm × 6 mm × 3 mm (115 mm was the grip-to-grip separation length, while the length of the narrow parallel section/inner diameter was 33 mm). The mold used to produce the bending test specimens was 60 mm × 10 mm × 1 mm according to the ISO 178:2019 [30] Standard. The conditions of the injection molding process are shown in Table 2. The conditions were the same for all 3 different types of molds, but they differed between samples.

All the results presented below were obtained by analyzing injection-molded samples in dimensions of 80 mm × 10 mm × 4 mm, except for the tensile and bending tests.

### 2.2. Thermal and Mechanical Properties

#### 2.2.1. Thermogravimetric Analysis and Differential Scanning Calorimetry Measurments

Thermogravimetric analysis (TGA) was carried out on all three samples using the TGA 2 STARe System (Mettler Toledo, Greifensee, Switzerland). Smaller samples were cut off (weighting about 20 mg) from the injection molded samples and measured in an N_2_ atmosphere with a gas flow of 20 mL/min between 25 and 650 °C at a heating rate of 5 °C/min. The dTG curves were calculated from obtained TGA curves.

Differential Scanning Calorimetry (DSC) was also performed on a small section of the main samples. The cut-off sections weighed between 10 and 12 mg and were sealed in aluminum pans. All the experiments were performed in an N_2_ atmosphere with a flow rate of 25 mL/min. First, heating was performed from −60 to 300 °C at a heating rate of 10 °C/min, then the samples were cooled down again to −60 °C at a heating rate of 20 °C/min and heated again from −60 to 300 °C at a heating rate of 5 °C/min. The DSC measurements were performed with the Differential Scanning Calorimeter DSC 3, STARe System (Mettler Toledo, Switzerland).

#### 2.2.2. Tensile Test

The tensile test was performed on an electromechanical testing machine, Vibrophore 100 (Zwick/Roel, Ulm, Germany). The test method is defined by the ASTM 638-22 Standard [29]. The test specimen is elongated until it breaks, and the associated force–displacement curve is recorded, from which the stress–strain curve can be calculated. The measurements were carried out with the speed of 1 mm/min at 23 °C and 50% relative humidity. Five repetitions were made for each material. Reduction in area (nominal)—the difference between the original cross-sectional area measured at the point of rupture after breaking and after all retraction has ceased, expressed as a percentage of the original area—was calculated according to Equation (1), where Z stands for reduction in area, $S_{0}$ for the original cross-sectional area and $S_{u}$ for the cross-sectional area at the point of rupture, measured after breaking the specimen.

(1) $$Z=\frac{S_{0}-S_{u}}{S_{0}}\times 100\%$$

After carrying out the tensile test, a Leica WILD M 10 microscope was used to determine the braking phases on the test specimens. The Digimizer software (version 6.3.0) was used to estimate the surface area of the individual breaking phase of each specimen.

#### 2.2.3. Bending Test

The 3-points bending test was performed on an electromechanical testing machine, Vibrophore 100 (Zwick/Roel, Ulm, Germany). The test method is defined by the ISO 178:2019 Standard [30]. The associated force–displacement curve was recorded, from which the stress–displacement curve was calculated. The measurements were carried out at 0.5 mm/min of speed, at 23 °C and 50% relative humidity. The distance between supports was 25 mm. Five repetitions were made for each material.

#### 2.2.4. Density Measurements 

The density of each material was determined on 5 of the 80 mm × 10 mm × 4 mm injection molded specimens according to the ISO 1183-1:2019 Standard [31]. An analytical balance KERN ABJ 320-4NM with an accuracy of ±0.0002 g (KERN & SOHN GmbH, Balingen, Germany) was used, along with a 50 mL liquid Hubbard pycnometer (Hofmann Glastechnik GmbH, Staudt, Germany). Ethylene glycol (Honeywell Riedel-de Haen, Seelze, Niedersachsen, Germany with ≥99.5% purity) was used as the liquid phase. The density of the materials was calculated according to Equation (2), where $\rho_{s}$ stands for the sample density, $m_{s}$ for the mass of the specimen, $\rho_{l}$ for the density of the liquid, $m_{\left(p+l\right)}$ for the mass of the pycnometer filled with liquid and $m_{\left(p+l+s\right)}$ the mass of the pycnometer filled with both liquid and sample.

(2) $$\rho_{s}=\frac{m_{s}\times \rho_{l}}{m_{\left(p+l\right)}+m_{s}-m_{\left(p+l+s\right)}}$$

### 2.3. Surface Properties

#### 2.3.1. Color Measurements

Samples r3L, rEL and rDSM were measured with the spectrophotometer Datacolor SF600 (Basel, Switzerland) (d/8 measurement geometry, with a measurement wavelength range from 400 nm to 700 nm and measurement area of 20 mm). The reflection measurements were calculated with the help of Datacolor Tools Plus (version: rev.2.8.3.29) software (Basel, Switzerland), resulting in the CIELAB values L*C*h and color differences dE* according to CIE 015:2018 [32].

#### 2.3.2. Contact Angle Measurements

The Surface Contact Angles (SCA) were measured, to estimate the surface wettability of the investigated materials via a goniometer, DataPhysics (Filderstadt, Germany). The Static Contact Angle (SCA) was measured using a drop of liquid resting on the surface. A small drop (5 μL) of Milli-Q water was placed carefully on the surface of the material. A goniometer with SCA 20 software was used to determine the SCA at room temperature. Five repetitions were made for each sample.

#### 2.3.3. Zeta Potential

The Zeta Potential measurements were performed with a SurPASS 3 (Anton Paar GmbH, Graz, Austria) using the adjustable gap cell with a flat surface for mounting the samples. A pair of each sample, with a size of 20 mm × 10 mm, was mounted on the sample holder using double-sided adhesive tape. The distance between the sample surfaces was adjusted to 100 ± 10 μm. The electrolyte solution was 1 mM KCl, and the pH was adjusted automatically with 0.05 M NaOH and 0.05 M HCl. The pH dependence of the Zeta Potential was determined in the range of pH 2.5–9. A pressure gradient of 200–600 mbar was applied to generate the streaming potential (Voltage).

#### 2.3.4. FTIR Spectroscopy and FTIR Microscopy Mapping

The infrared spectra were recorded with a PerkinElmer Spectrum 3 ATR-FTIR spectrometer (PerkinElmer, Waltham, MA, USA) The GladiATR accessory, (PIKE Technologies, Madison, WC, USA) contained a monolithic diamond crystal. A total of 16 scans with a resolution of 4 cm^−1^ were acquired from each sample. All the spectra were recorded at room temperature in a wavenumber range between 4000 and 600 cm^−1^.

**Figure 1:**
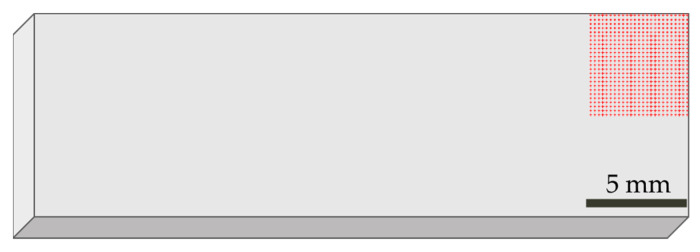
Investigated area of the surface with determined markers.

The FTIR microscopy mapping was performed using the FTIR PerkinElmer Spectrum 3 with the Spotlight 200i frontier microscope (PerkinElmer, Waltham, MA, USA). The mapping was performed on the surface of the injection-molded samples at sizes of 5 × 5 mm for each material produced. On the examined surface a mesh combination of 25 × 25 markers was determined with 100 µm spacing between them (as shown in Figure 1). The size of the aperture was set to 100 × 100 µm. The measurements were performed in ATR mode with a diamond ATR crystal using 18% of force. A total of 16 scans with a resolution of 4 cm^−1^ were acquired from each sample. All the spectra were recorded at room temperature in a wavenumber range between 4000 and 700 cm^−1^. The results obtained were analyzed using the SpectrumIMAGE software (version: R1.11.2.0016) from PerkinElmer. For samples r3L and rDSM, the spectra obtained on the surface were compared with the PP spectra, and for sample rEL with the PA 6 spectra.

### 2.4. Morphology

#### 2.4.1. X-ray Diffraction

The crystalline structure of the samples was assessed using a Bruker D2 Phaser Diffractometer (Bruker, Billerica, MA, USA) (Cu–Kλ radiation; 1.5406 Å). The measurements were performed with a step size of 0.03° and a time/step of 0.2 s. Cut pieces (10 mm × 10 mm × 4 mm) of the samples were placed on a Si zero background sample holder, and the XRD spectra were collected in the 2θ range from 5° to 70°.

#### 2.4.2. Stereomicroscopy

A digital microscope, the Keyence VHX-7100 (Keyence Corp., Itasca, IL, USA), was employed for the examination of the macrostructure of the cryofracture surfaces on the transversal cross-section. The fracture surfaces on the transversal cross-section were prepared by fracturing the samples under cryogenic conditions (liquid nitrogen). The specimens were immersed in liquid nitrogen for 2 min, and then broken in the middle to obtain a cross-section for the stereomicroscopy.

#### 2.4.3. FTIR Microscopy Mapping of the Cross-Sections

**Figure 2:**
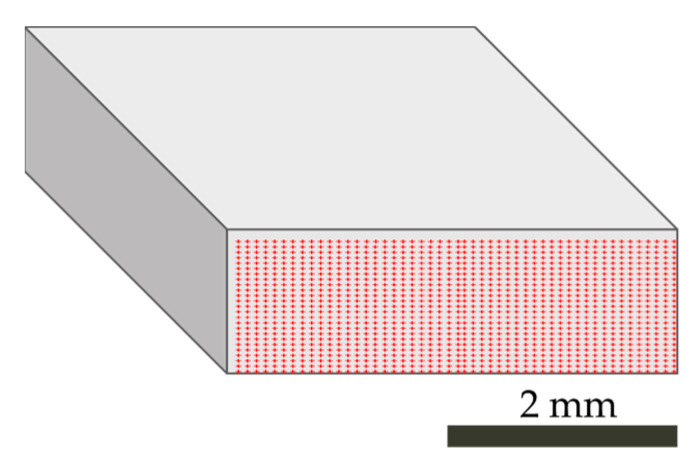
Investigated area of breaks with determined markers.

The FTIR microscopy mapping was performed using the FTIR PerkinElmer Spectrum 3 with the Spotlight 200i frontier microscope (PerkinElmer, Waltham, MA, USA). The mapping was performed on the surfaces of the breaks previously investigated with stereo microscopy. A mesh combination of 20 × 50 markers was determined with 100 µm spacing between them (as shown in Figure 2). The size of the aperture was set to 100 × 100 µm. The measurements were performed in ATR mode with a diamond ATR crystal using 18% of force. A total of 16 scans with a resolution of 4 cm^−1^ were acquired from each sample. All the spectra were recorded at room temperature in a wavenumber range between 4000 and 700 cm^−1^. The obtained results were analyzed using the SpectrumIMAGE software from PerkinElmer. For the r3L sample, the spectra obtained on the surface were compared with PP spectra, for the rEL sample with PA 6, and for the rDSM sample again with PP spectra.

## 3. Results

### 3.1. Optical Evaluation of Test Samples

**Figure 3:**
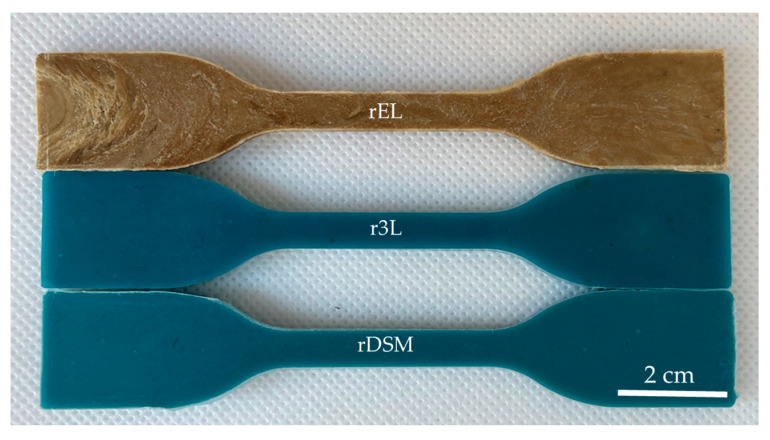
Photography of the bending tests specimens from all three materials.

After the injection molding of each material, all the specimens were observed and compared, as shown in Figure 3. The visual assessment of all the produced specimens revealed that no fibers were visible on the surface. When comparing the samples, the color difference between rEL and r3L or rDSM was clearly visible, while the difference between r3L and rDSM was not so obvious. The color of rEL was light brown, while r3L and rDSM were both blue in color, the only difference being that the rDSM samples were slightly lighter. The surface of all three materials was nice and smooth, and no irregularities could be seen on the surface.

### 3.2. Thermal and Mechanical Properties

#### 3.2.1. Thermogravimetric Analysis and Differential Scanning Calorimetry

**Figure 4:**
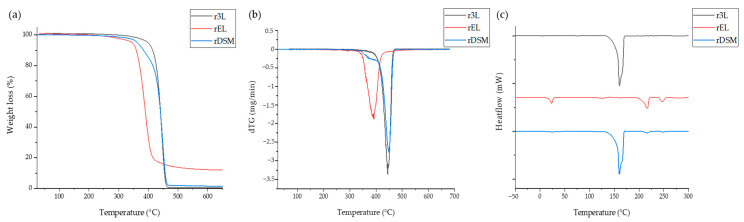
(**a**) Thermogravimetric results of all three materials, (**b**) dTG curves, (**c**) Differential Scanning Calorimetry thermograms of all three materials.

Figure 4a shows the thermograms of mass loss as a function of temperature, which was monitored to evaluate the thermal properties of the recycled polymers. It can be seen that the thermal stability of rEL was lower than that of r3L. The degradation of the rEL started faster, at around 300 °C, while the degradation of r3L started at a temperature above 400 °C. The combination of those materials affects the thermal stability so that the thermal degradation of rDSM starts at a lower temperature, but also at a lower rate than r3L. The mixture of materials from ear loops combined in the rEL material have different thermal properties and can be seen from the diagram. The degradation starts at about 300 °C, as is known for PU [33,34], at about 400 °C the degradation of PA 6 starts, and ends at 470 °C [35], when a plateau starts and lasts until 650 °C, which is known for PET [36], for which the last 15–17% of weight loss usually occurs in a temperature range higher than 650 °C. As can be seen from the dTG curves in Figure 4b, the decomposition was carried out as a one-step process for the r3L and rEL materials, and a two-step process for the rDSM material. According to Figure 4b, from the dTG curves, the highest decomposition rates were found to be at 445 °C for r3L and 400 °C for rEL. The presence of rEL materials in the rDSM sample can also be seen from the dTG curve, where the first lower signal appeared in the same range as for rEL. The highest decomposition rate for rDSM was found to be at 450 °C, and, as coincided with the decomposition rate of r3L. The signals on the Differential Scanning Calorimetry thermograms shown in Figure 4c, represent the melting points of the materials and confirm the diversity of the materials in the rEL samples. The melting point of r3L is at a temperature of about 160 °C, the known melting point temperature of PP [37]. Several signals were determined for rEL, of which 220 °C is the melting point of PA 6 [38] and 250 °C refers to PET [39]. Furthermore, a lower signal were detected at about 20 to 30 °C, which can be attributed to the presence of elastane [16,40]. All the signals detected on the r3L and rEL curves can also be detected on a curve of the rDSM sample, whereby the signals assigned to PU, PA 6 and PET are less intense than the signal for PP, which is consistent with the weight ratio of the materials.

#### 3.2.2. Tensile Test

**Figure 5:**
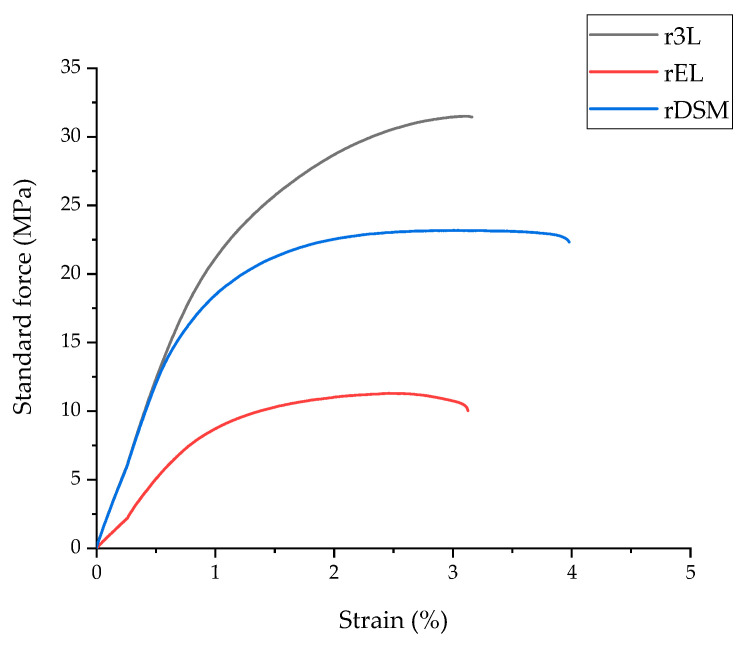
Stress–strain curves from the tensile tests of all three materials.

**Figure 6:**
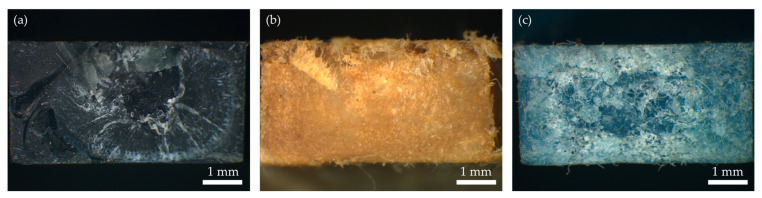
Microscopic images of the breaks caused by the tensile test analysis of (**a**) r3L, (**b**) rEL and (**c**) rDSM.

The graph in Figure 5 shows the tensile test properties of all three samples. The results are represented as standard force in MPa as a function of strain in %. As is shown in the graph and in Table 3, the r3L sample withstands maximum stress and has the higher Young’s modulus, which means that this sample is more rigid than the other two. The rEL sample is less rigid as it has the lowest Young’s modulus, and it also withstands the lowest maximum stress. The combined material rDSM has a greater capacity for plastic deformation, which is determined by the highest strain at break. This could be due to the fibers from the ear loops present in the PP matrix visible in Figure 6c. Comparing the r3L and rDSM results to similar copolymers [41] and recyclates obtained by Battegazzore et al. [16], it was found that Young’s modulus and withstand maximum stress at break of r3L and rDSM are significantly higher, while elongation at break is slightly lower. This low elongation at break behavior is similar to what is found for commercial recycled materials (e.g., 10% for EKOBOARD Recycled by Ekon B.V., Born, The Netherlands). When comparing the results of rEL to similar recyclate obtained by Battegazzore et al. [16], it can be found out that the maximum stress is slightly higher and Young’s modulus is slightly lower, while the elongation at break is significantly lower for the rEL material.

Figure 6 shows microscopic images of the breaks caused by the tensile test analysis. Figure 6a shows the break of r3L and shows clearly the break in three phases. The first phase of the break occurred at the edges, where the surface is flat, and it represents 34.7% of the break surface. The second phase was the pulling phase and can be seen as an elliptical, pulled-out center of the sample; it represents about 58% of the break surface. The third and final phase of the break can be seen in the exact center of the break with a flat surface. It was caused by a similar shear force as in the first phase of the break and represents 7.3% of the broken area. Figure 6b shows the break of the rEL specimens. The break only occurred in two phases. The first phase can be seen in the center and represents 64.3% of the broken area. The surface is flat and was caused by shear force, while the second phase occurred at the edges of the sample where pulled-out fibers can be seen and represents 35.7%. Figure 6c shows a similar break in three phases of rDSM, as in the case of r3L. The main difference is the visible pulled-out fibers, which also contributed to a higher elongation at break rate. The first phase covers 46% of the break area, the second phase covers 33% and the third phase covers 21% of the broken area [42].

#### 3.2.3. Bending Test

**Figure 7:**
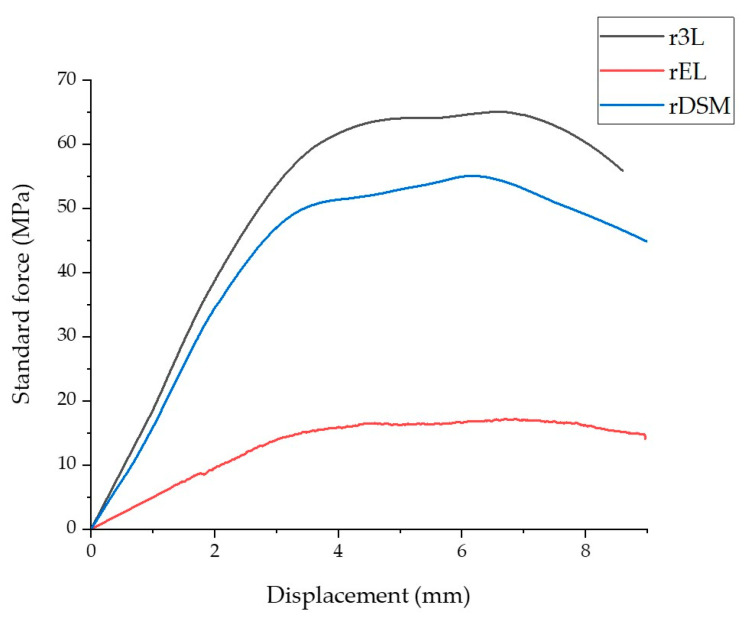
Stress–strain curves from the bending tests of all 3 materials.

The graph in Figure 7 shows the bending test properties of all three samples. The results are presented as standard force in MPa as a function of strain in %. The results obtained are shown in Table 4 and are similar to the results of the tensile test. As can be seen from the graph and Table 4, the r3L specimen again withstands the maximum stress and has the higher flexural modulus. The rEL sample again had the lowest results, the lowest flexural modulus, and withstood the lowest maximum stress. The bending test results of the combined material rDSM are showing that the flexural modulus and the maximum stress were reduced by the addition of ear loops materials to the three-layer matrix (PP). Compared to untreated PP, the flexural modulus of r3L and rDSM are lower (untreated PP—2580 MPa [43]), while the maximum stress is higher for r3L and lower for rDSM (untreated PP—58.20 MPa [43]).

#### 3.2.4. Density Measurements

The densities of all three materials obtained are listed in Table 5. If the measured densities of r3L and rDSM are compared with those of untreated PP, it can be seen that the density of r3L is in the range of the characteristic PP density (0.85–0.92 kg/m^3^ [44,45]), while rDSM is slightly above this range. The increase in the density of the rDSM material is due to the added ear loop material. rEL had the highest density of all three samples, which was expected, since all the materials mixed in rEL PA6 (characteristic density 1.12–1.15 kg/m^3^ [44]), PU (characteristic density 1.20–1.26 kg/m^3^ [44]) and PET (characteristic density 1.38–1.41kg/m^3^ [44]) had higher density than the rDSM and r3L materials.

### 3.3. Surface Properties

#### 3.3.1. Colorimetric Analysis

**Figure 8:**
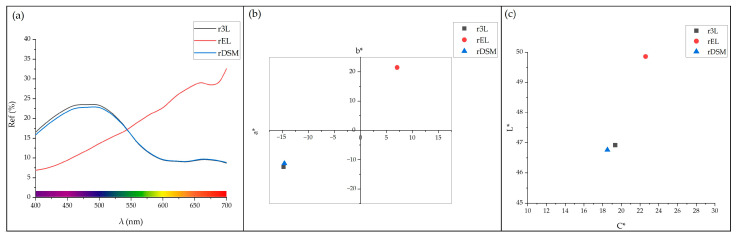
(**a**) Reflection curves of all three samples, (**b**) Color characteristics of all three samples in the CIE color space, and (**c**) Color characteristics of all three samples in the CIE L*/C* diagram.

The colors of the samples were determined by colorimetric analysis. As already determined during the visual inspection, the colors of the r3L and rDSM samples were blue. This is also evident from the results of the colorimetric analysis, which are shown in the diagrams in Figure 8a,b. The maximum reflectance of these two samples was in the range of 450 to 500 nm, i.e., in the range of the violet and blue colors. As can also be seen in Figure 8b in the CIELAB color space, these two r3L and rDSM samples lie in the third quadrant, which represents green and blue colors. Visual inspection of rEL revealed that the sample had a brown color, while the colorimetric analysis showed that this sample lies in the first quadrant (range of red and yellow colors), as shown in the diagrams in Figure 8a,b. When ear loops were added to the three-layer part of DSM in the combined sample rDSM, the reflectance curve of rDSM changed slightly compared to r3L, between 400 and 510 nm, from 510 to 700 nm, the two curves then overlayed. Figure 8c shows the CIE L*/C* diagram for all three samples. From this diagram and the values from Table 6, it can be seen that there was a slight difference in saturation between the samples (C*), the saturation of rEL was 22, while the saturation of rDSM and r3L was about 19. For lightness (L), there was a smaller difference between rEL and the other two samples, while there was practically no difference between r3L and rDSM. It was found that adding ear loop material to the three-layer part of DSM had no effect on the colors of rDSM patterns. As also shown in Table 6, the color difference (ΔE*) was only 1.16. The small effect of the ear loops material on the color of the rDSM sample was due to the small proportion (14%) of this material in the rDSM, and, as will be shown in the continuation of the article, the majority of the ear loops’ material was located in the middle of the test samples, while the colorimetric analysis was performed on the surface.

#### 3.3.2. Contact Angle Measurements

**Figure 9:**
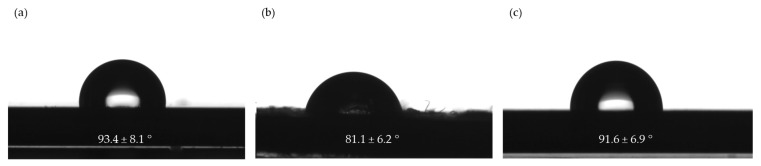
Water drop and Contact Angles on the surface ± Standard Deviation of (**a**) r3L material, (**b**) rEL material and (**c**) rDSM material.

Changes in the surface properties of plastics, when different polymers are combined, can be monitored by Contact Angle (CA) measurements in relation to the hydrophobicity/hydrophilicity of the surface [46]. The CA measurements were carried out on the r3L, rEL and rDSM samples. As can be seen in Figure 9, r3L was the most hydrophobic material, while rEL was hydrophilic. When both materials were combined in the rDSM sample, the presence of ear loops’ materials caused a slightly lower CA. The data for the CA of a water drop on the surface of virgin PP varied from 76° to 104.9° [47,48,49]. The r3L material and rDSM material results of CA measurements are comparable to the results of the virgin PP and are above 90°, which means that they are hydrophobic. The results of the rEL CA measurements showed values below 90°. Because the PU and PET are hydrophobic (higher CA values than 90°), and only PA 6 is hydrophilic (lower CA values than 90°), we can conclude that the main influence on the surface area of the rEL results was caused by PA 6 [47,50]. This was additionally proven by the FTIR mapping of the surface presented in Section 3.3.3.

#### 3.3.3. Zeta Potential

The Zeta Potential (ZP) indicates the charge present on the surface of the solid material. If the ZP is positive, it suggests that the surface is positively charged, while a negative ZP indicates a negatively charged surface [51].

ZP influences the interactions between solid materials and the surrounding molecules or particles in the liquid medium. Monitoring the ZP of recycled materials is crucial for understanding their surface properties and reactivity. By assessing the ZP, researchers can gain insights into how these materials interact with their environment and other substances, which is essential for optimizing recycling processes and identifying potential applications. That is why it is extremely important to monitor the ZP of recycled materials, as it may provide insight into the materials ionic characters and, furthermore, their reactivity and ability to bind specific substances.

**Figure 10:**
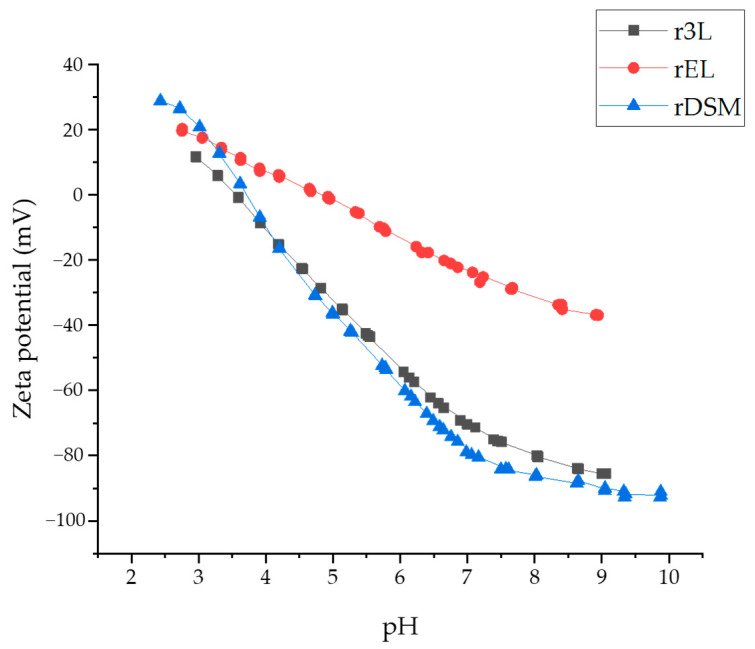
Surface zeta potential as a function of pH of all three obtained materials.

Samples r3L and rDSM exhibited very similar charging behavior. Both samples demonstrated an isoelectric point at around pH = 4.5, indicating that the surface of the materials was acidic. It is known for both samples that PP is the dominant fraction: fully in r3L and overall, but not entirely, in rDSM. Therefore, both ZP curves (Figure 10) for these two samples are in accordance with the theoretical PP curves known to act as hydrophobic materials, where the isoelectric point falls within a pH range of 4 to 6, varying based on specific conditions [52].

Above the isoelectric point, the surface of PP may carry a slight negative charge due to the adsorption of hydroxyl ions (−OH) or other anionic species present in the solution, or due to the deprotonation of weak acidic functional groups. At lower pH values (below the isoelectric point, acidic conditions), the adsorption of oxonium ions may occur, or protonation of surface functional groups may arise, resulting in a reversal to a positive charge. The phenomena of protonation and deprotonation may be the case for rDSM, where some small fractions (around 14%) of PET, PA6, and PU may be present, possessing ionizable groups.

The anionic character of samples r3L and rDSM was observed through the negative ZP above the isoelectric point, and it was evidenced by a relatively low plateau level of negative ZP (i.e., lower than −80 mV). This suggests a tendency to attract positively charged species in this pH region.

Conversely, sample rEL was less acidic, with an isoelectric point shifted towards a higher pH, specifically at pH = 5.7. This sample comprised a mixture of multiple polymeric materials, including PET, PU and PA 6. As previously predicted, PA 6 was prominent in this mixture, which may suggest that the higher isoelectric point of this sample was due to the presence of weak basic amino groups in PA 6.

The variances in the negative plateau of the ZP among all the samples may also be attributed to differences in the surface hydrophilicity and hydrophobicity. If the sample’s surface becomes less hydrophobic, the negative ZP plateau values decrease. For example, rEL exhibited a less negative plateau level of ZP, consistent with a lower CA and a more hydrophilic nature (being the only hydrophilic sample among them all), if surface chemistry is not considered.

#### 3.3.4. FTIR Spectroscopy and FTIR Microscopy Mapping

**Figure 11:**
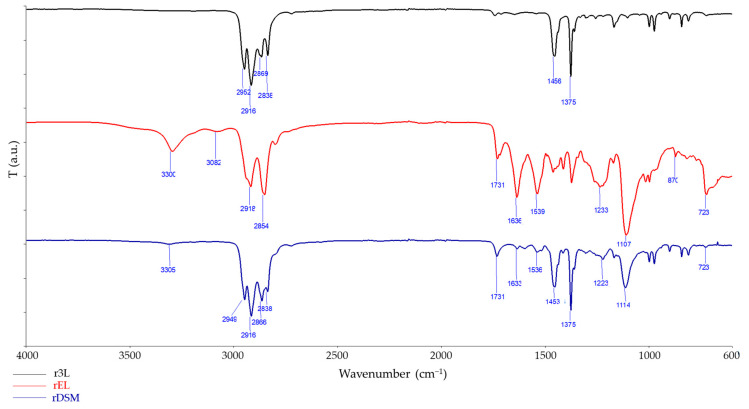
Infrared spectra scanned on the surface of all three materials.

Further surface analysis was carried out using an FTIR spectrometer with ATR. The measurements were performed on all sides of the test samples and at multiple points. No changes were detected between multiple FTIR scans of the same material. The FTIR spectra obtained are shown in Figure 11. The spectra of r3L are characterized by the typical transmittance of PP [16,53]. The characterization of materials from the rEL spectra was much more complicated. As we have already noted in the literature and in the explanation of the thermal properties of the obtained materials, there are three different materials used to produce ear loops: PU, PA 6 and PET. For each of these materials, specific signals can be detected in the FTIR spectra of the rEL sample. The signals detected at 2918 cm^−1^ and 2854 cm^−1^ are characteristic of all three materials, as they represent the C-H stretching of the aliphatic group [16,54]. The presence of PU was demonstrated by the detection of N-H bonds at 3300 cm^−1^, which is one of the characteristics of urethane groups [16,55]. Two other specific signals can be detected at 1107 cm^−1^ (C-O bond stretching) and at 1233 cm^−1^ (C-N bond stretching) [16,54,56]. Another characteristic signal indicating the presence of PU is at 1731 cm^−1^ where the band characteristic of C=O stretching can be found [57]. The presence of PA 6 was detected by identifying signals at 1636 cm^−1^ and 1539 cm^−1^ [16,58]. The presence of PET was detected by the signals at 1731 cm^−1^ and 1233 cm^−1^, which can also be assigned to PU, but the signals at 870 cm^−1^ and 723 cm^−1^ (the presence of C–H on the aromatic rings), which are in the specific fingerprint region of FTIR, are clear evidence for the presence of PET [16,59]. The spectra of rDSM are characterized by the typical transmission signals of PP in the range from 2949 to 2838 cm^−1^, at 1453 cm^−1^ and at 1375 cm^−1^ with high intensity, while the signals characteristic of rEL at 3305 cm^−1^, 1731 cm^−1^, 1636 cm^−1^ and 723 cm^−1^ are slightly shifted and of a lower intensity as this material corresponded to a small amount in the mixture (rEL accounted for only 14% of the mass fraction in the rDSM material).

**Figure 12:**
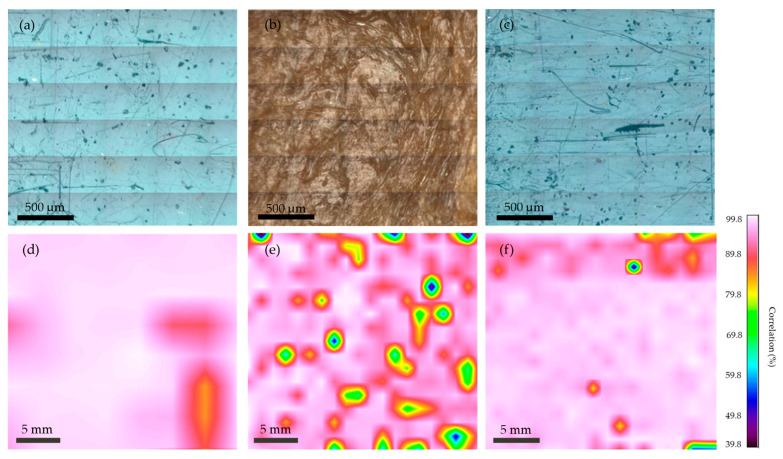
Optical images of the surface of all three materials made by the FTIR microscope and associated FTIR mapping results: (**a**) Optical image of r3L, (**b**) Optical image of rEL, (**c**) Optical image of rDSM, (**d**) Mapping of r3L, (**e**) Mapping of rEL and (**f**) Mapping of rDSM.

Further analysis was performed by FTIR mapping using micro-FTIR. With this method, we obtained the results of the distribution of the different materials on the surface of the polymer blends or composites. The resolution of the results was in the microscale range. The mapping results are shown in Figure 12, with the corresponding microscope images of the examined surface. Figure 12a shows a microscope image of the surface of r3L. The mapping results of this surface are shown in Figure 12d. This analysis revealed a correlation with the PP spectra. As can be seen, the correlation was over 95% and was distributed evenly over the entire surface. There are two red spots that show a correlation of about 90%, due to the lower intensity of the obtained spectra and not due to different materials or impurities. Figure 12b,e shows a microscope image of the surface and the mapping result for the rEL material. The FTIR spectra scanned over the entire surface were compared with the spectra of PA 6. The result shows that PA 6 was the main material on the surface, on which the blue and green spots are distributed evenly. These spots indicate the presence of PET and PU material. Figure 12c shows a microscope image of the surface of rDSM. The mapping results of this surface are shown in Figure 12f. The spectra obtained were compared with the spectra of PP. The white, pink and red colored surface indicates the presence of PP, while the green, blue and yellow spots represent the presence of the ear loop materials. This result proves that the ear loop materials are also present on the surface of the rDSM, even if they were not visible under a microscope.

### 3.4. Morphology

#### 3.4.1. X-ray Diffraction

**Figure 13:**
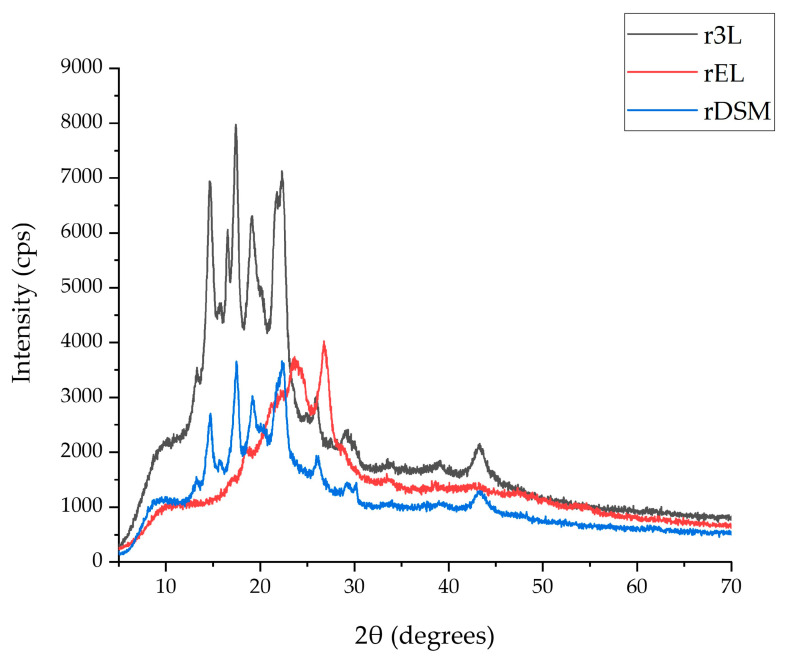
XRD graphs of all three materials.

The wide-angle X-Ray Diffraction spectra of the prepared recycled materials are presented in Figure 13, including analysis of the peak positions, their intensity and the appearance of the background hump can assist in identifying the phases contained within the samples, their concentration and organization, as well as the extent of the amorphous phase, respectively. The diffractogram for the sample was produced from the main component of the facemask, namely the nonwoven/melt-blown three-layer structure (sample r3L), which exhibited the peaks characteristic of the α-monoclinic PP, with slight shifts: 16.5°; 17.4°; 19.2°; 22.3°; 26.1° and 29.1°, while the peaks at the 14.7° and 21.9° angles refer to a mesomorphic phase [60,61]. Additionally, the occurrence of a peak at 15.9° is assigned to the β-hexagonal structure of PP, resulting in superimposed spectra due to the appearances of several phases. The XRD spectra of the mask layers prior to extrusion exhibited the majority of the above peaks, with the exception of the 16.5° peak, which corresponded to the (040) crystallographic plane. Again, slight shifts of the peak positions in the spectra of the non-extruded and extruded r3L samples are noticeable, which we can ascribe not only to the thermal processing and extrusion of the nonwoven/melt-blown layers, but also to the different morphology of the examined samples (fibrous membranes vs. injection-molded specimens). The XRD spectra for sample rEL showcases additive contributions from the components of the ear loop pieces; the peak at 22.7° can be attributed to the (002) crystallographic plane of PA6 [62], while the sharp peak at 26.5° corresponds to the (100) plane of PET [63]. The pronounced amorphous character of sample rEL was due to the presence of the PU in the starting material; PU itself exhibited a broad peak at about 2θ = 19° [64]. The diffractogram of the rDSM sample reflects clearly the composition of this particular extruded material; prevalent amounts of nonwoven/melt-blown layers, i.e., PP, dominated the spectra, and it resembles the r3L sample closely. The most significant difference of this hybrid sample, when compared to r3L, is the lower intensity of the peaks, which indicates a decrease in the crystallinity of the material (all the sample specimens were prepared in an identical manner with the same masses, therefore allowing us to attribute the peak intensity directly to the extent of the crystalline organization). The diffrac.Eva (Bruker) software (version number 5.1.0.5) was used to calculate the degree of crystallinity of the extruded materials: sample r3L possessed 38.7% crystallinity, while rDSM showcased 31.1%. This decrease in overall crystallinity can be attributed to the presence of the far less structurally organized sample, i.e., rEL, with a calculated degree of crystallinity of 25.4%. As noted above, the ear loop pieces contained PU, which is characteristically amorphous, and thus can contribute greatly to the decrease in overall crystallinity.

#### 3.4.2. Stereomicroscopy

**Figure 14:**
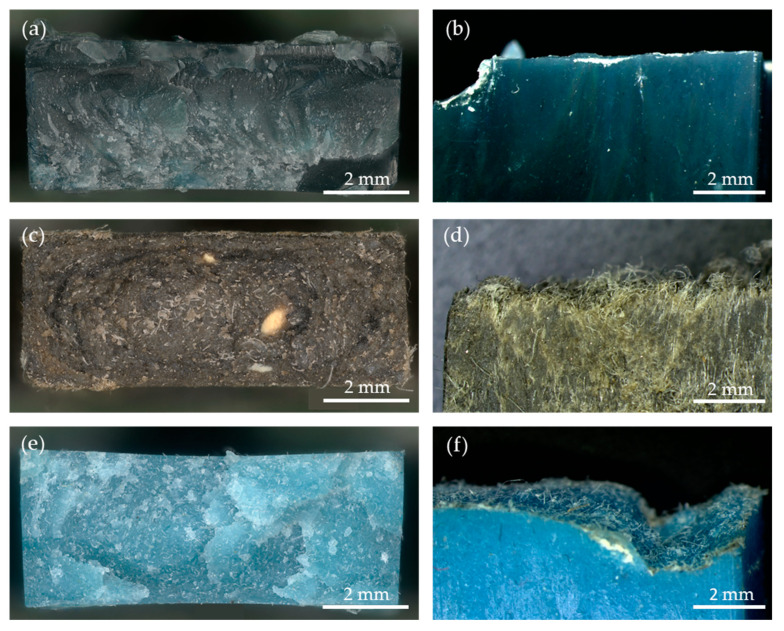
Stereomicroscopy images of cross-sections of all three samples from two different angles: (**a**) r3L surface of cross-section, (**b**) r3L edge of cross-section, (**c**) rEL surface of cross-section, (**d**) rEL edge of cross-section, (**e**) rDSM surface of cross-section, and (**f**) rDSM edge of cross-section.

**Figure 15:**
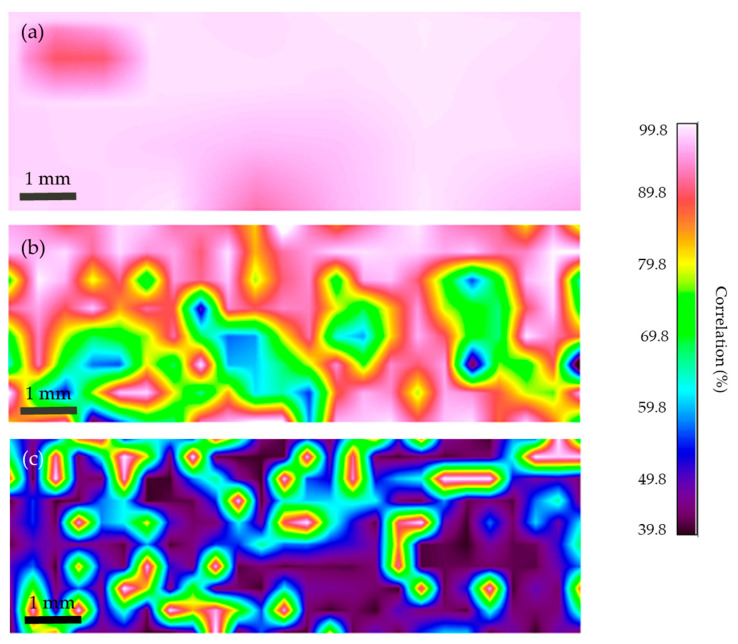
FTIR mapping results of the cross-section surfaces shown in Figure 14, (**a**) FTIR mapping of the r3L cross-section, (**b**) FTIR mapping of the rEL cross-section and (**c**) FTIR mapping of the rDSM cross section.

Similar to the stereomicroscopic images of the tensile test breaks, we obtained another cross-section image by fracturing the samples using liquid nitrogen to exclude the influence of the direction of force on the formation of the fracture surface. Figure 14a shows the surface of the cross-section of the r3L material, while Figure 14b shows its edge. As can be seen from the images, there is a flat surface on which no particles or unmelted fibers can be seen. This means that the melting process during extrusion and injection molding was successful and resulted in a homogeneous material. Figure 14c shows the surface of the cross-section of rEL and Figure 14d shows the edge. In these two images, a high number of unmelted fibers can be seen, which was due to the presence of fibers with a higher melting point temperature (PU and PET [16,40]) than the set process temperature. It can also be seen that the orientation of the fibers was in the direction of the injection molding process. The cross-section of the rDSM material is shown in Figure 14e, while Figure 14f shows its edge. This sample is the most interesting, as it shows clearly a flat surface of molten PP with enclosed unmelted fibers, which are again oriented in the direction of the injection molding process. The homogeneous distribution of the fibers in the polymer matrix was also confirmed by the FTIR image of the cross-section shown in Figure 15c. As already mentioned in Section 3.2.2, the presence of fibers in this material leads to higher values of elongation at break, which means that the material can withstand higher plastic deformation.

#### 3.4.3. FTIR Microscopy Mapping of the Cross-Sections

In order to determine the distribution of the different materials in the center of the injection molded samples, FTIR mapping was performed on the cross-sections of the previously stereomicroscopically examined surfaces, which are shown in Figure 14. Figure 15 shows the results of the mapping for each sample. Pure PP was detected on the entire surface of the r3L cross-section, as shown in Figure 15a. Figure 15 shows the mapping result for rEL, obtained comparing the correlations with the PA 6 spectra. As can be seen, the pink and white spots are located mainly at the edges and represent the distribution of PA 6. In the middle of the test samples, where green and blue spots can be seen, are the materials PET and PU. These results also confirm the conclusions of the Contact Angle and Zeta Potential measurements, according to which PA 6 was distributed mainly on the surface of the samples, while the other two materials were located in the center. Comparing these results with the mapping of the surface of rEL (see Figure 12e), a significantly higher proportion of PET and PU was found. Figure 15c shows the results for the rDSM material. In this map, the darker colors—black and blue—represent PP, while the lighter spots represent the unmelted fibers (PA 6, PET, PU) from the ear loops. Comparing this result with the mapping of the surface of rDSM (see Figure 12f), it can be seen that the materials from the ear loops were located mainly in the center of the samples, where they were evenly distributed, while they were less present on the surface. This finding was further supported by the results of the Contact Angle Zeta Potential, and Colorimetric measurements at the surface, where it was found that the results between rDSM and r3L (PP) did not differ significantly.

## 4. Conclusions

To summarize, the focus of this manuscript was on the detailed analysis of the recyclates of DSM obtained by the thermomechanical process. A detailed analysis of the three different materials obtained from DSM (r3L, rEL and rDSM) was carried out, including not only thermal and mechanical tests, but also morphological and surface tests, in order to understand the distribution of the different polymers when they are processed together thermo-mechanically and their influence on the mechanical and surface properties. This work complements and extends the previously published results of Battegazzore et al. [16]. These analyses led us to several interesting conclusions: In terms of mechanical properties, the addition of fibers from ear loops into the PP melt from a three-layer part leads to lower stiffness, but also to higher toughness of the material. Regarding the morphology of the samples, it was found that the PA 6 in the rEL material was distributed mainly on the surface of the produced samples, while the PET and PU materials were distributed in the center of the test samples. When these three materials were added to the PP melt, it was found that all three materials were distributed mainly in the center of the samples, while the surface of the material was mainly from PP. Compared to the virgin PP, r3l and rDSM showed high potential, as they had similar mechanical and surface properties to the virgin material (PP). As also stated by Battegazzore et al. [16], some improvements in the performance of recycled materials would be necessary. The data obtained in this study provide a good starting point for future research in the search for suitable additives, fillers or compatibilizers. A new functional material with added value can be obtained by adding additives, fillers or compatibilizers. If the additives are natural biopolymers or polyphenols, a new recyclate with antimicrobial properties can be produced from such a material.

In this way, we would introduce the concept of a circular economy for the handling of disposable surgical masks (DSMs), whose improper handling has a major negative impact on the environment and whose recyclate can be used to produce new personal protective equipment (PPE) with added value.

## Figures and Tables

**Table 1:** Extrusion conditions.

Sample [Material] (Abbreviation)	Temperature Zones (°C)								Screw Speed (RPM)
	Die	8	7	6	5	4	3	2	
Recycled three-layered part of the DSM [PP] (r3L)	165	170	180	190	200	195	190	185	125
Recycled ear loops [PET, PU and PA6] (rEL)	235	230	230	230	225	220	220	210	50
Recycled combined materials [86% r3L and 14% rEL] (rDSM)	165	170	180	190	200	195	190	185	125

**Table 2:** Injection molding conditions.

Sample	Cylinder Temperature (°C)	Mold Temperature (°C)	Injection Pressure (bar)	Injection Time (s)	Post Injection Pressure (bar)	Post Injection Time (s)
r3L	180	65	600	5	450	5
rEL	240	70	500	5	450	5
rDSM	180	65	600	5	450	5

**Table 3:** Average values of Young’s modulus (E), maximum stress ($\sigma_{M}$), strain at break ($\varepsilon_{B}$) and reduction in area (Z) from the tensile tests of all three materials with determined ± Standard Deviation (STD).

Material	Average E (MPa) ± STD	$\mathrm{Average}\;\sigma_{M}$ (MPa) ± STD	$\mathrm{Average}\;\varepsilon_{B}$ (%) ± STD	Average Z (%) ± STD
r3L	2339.5 ±8 5.6	30.5 ± 1.7	3.2 ± 0.2	2.9 ± 0.2
rEL	858.2 ± 56	10.7 ± 0.4	3.1 ± 0.2	0.2 ± 0.03
rDSM	2150.7 ± 71.5	22.7 ± 0.8	4.1 ± 0.4	2.2 ± 0.17

**Table 4:** Average values of flexural modulus (E_f_), maximum stress ($\sigma_{M}$) and maximum strain ($\varepsilon_{M}$) from the tensile tests of all three materials ± Standard Deviation (STD).

Material	Average E_f_ (MPa) ± STD	$\mathrm{Average}\;\sigma_{M}$ (MPa) ± STD
r3L	1927.2 ± 80.1	63.8 ± 4.3
rEL	439.9 ± 61.4	16.4 ± 1.9
rDSM	1655.3 ± 60.7	54.7 ± 1.3

**Table 5:** Average values of density for all three materials.

Sample	Average Density (kg/m^3^) ± STD
r3L	0.905 ± 0.002
rEL	1.156 ± 0.014
rDSM	0.969 ± 0.004

**Table 6:** Colorimetric values (D65/10) of all three samples.

Sample	L*	a*	b*	C*	Hue	ΔE*
r3L	46.92	−14.81	−12.45	19.35	220.05	/
rEL	49.86	7.06	21.46	22.59	71.80	/
rDSM	46.76	−14.65	−11.31	18.51	217.67	1.16

## Data Availability

Data can be provided by the corresponding author on request (due to privacy).
